# Characterizing memory loss in patients with autoimmune limbic encephalitis hippocampal lesions

**DOI:** 10.1002/hipo.23150

**Published:** 2019-08-31

**Authors:** Meher Lad, Sinéad L. Mullally, Alexandra L. Houston, Tom Kelly, Timothy D. Griffiths

**Affiliations:** ^1^ Institute of Neuroscience Newcastle University Newcastle upon Tyne United Kingdom; ^2^ The Neuropsychology Department Royal Victoria Infirmary Newcastle upon Tyne United Kingdom; ^3^ Wellcome Trust Centre for Neuroimaging University College London London United Kingdom

**Keywords:** episodic memory, hippocampus, recall memory, recognition memory, retrograde‐amnesia, semantic memory, voltage‐gated potassium channel complex antibody associated limbic encephalitis (VGKCC‐Ab LE)

## Abstract

Since the publication of Scoville and Milner's (1957) seminal paper, the precise functional role played by the hippocampus in support of human memory has been fiercely debated. For instance, the single question of whether the hippocampus plays a time‐limited or an indelible role in the recollection of personal memories led to a deep and tenacious schism within the field. Similar polarizations arose between those who debated the precise nature of the role played by the hippocampus in support of semantic relative to episodic memories and in recall/recollection relative to familiarity‐based recognition. At the epicenter of these divisions lies conflicting neuropsychological findings. These differences likely arise due to the consistent use of heterogeneous patient populations to adjudicate between these positions. Here we utilized traditional neuropsychological measures in a homogenous patient population with a highly discrete hippocampal lesion (i.e., VGKCC‐Ab related autoimmune limbic encephalitis patients). We observed consistent impairment of recent episodic memories, a present but less striking impairment of remote episodic memories, preservation of personal semantic memory, and recall but not recognition memory deficits. We conclude that this increasingly well‐characterized patient group may represent an important homogeneous population in which the functional role played by the hippocampus may be more precisely delineated.

1

The human hippocampus literature is abound with contentious debates and conflicting evidence surrounding the precise role played by the hippocampus in support of memory. Key areas of debate include the hippocampus' role in recent versus remote memories (for recent reviews see Squire, Genzel, Wixted, & Morris, 2015; Sekeres, Winocur, & Moscovitch, 2018), in autobiographical versus semantic memories (for recent review see Dede & Smith, 2016), and in recollection‐ versus familiarity‐based retrieval (for recent review see Aggleton & Morris, 2018).

With respect to the former of these debates, and in a comprehensive review of the literature from 1957 to 2010, Winocur, Moscovitch, and Bontempi (2010) reported roughly equal number of cases where hippocampal damage was either (a) associated with either nongraded or temporally extensive retrograde amnesia or (b) resulted in the classic temporally graded retrograde amnesia described by the Standard Consolidation (SC) model (e.g., Squire, 1992). Proponents of SC model have attributed the source of this variation to lesions selectivity, whereby ungraded retrograde amnesia is a consequence of extra‐hippocampal damage. On the other side, those who argue in favor of ungraded‐retrograde amnesia contend that traditional tasks of remote retrograde memory are not sensitive enough to detect subtle differences between the recall of true remote autobiographical memories and more schematic versions of such memories (for review see Winocur & Moscovitch, 2011). Hence, according to this perspective, traditional findings of preserved remote memories are likely to be an artifact of limited methodologies.

Relatedly, the neuropsychological evidence for and against a unified role for the hippocampus in both semantic and episodic memory is complex (Winocur et al., 2010; Winocur & Moscovitch, 2011). As SC model does not distinguish between the role played by the hippocampus in support of different forms of declarative memories (e.g., between episodic/context‐dependent memories and semantic/context‐general memories), neuropsychological cases and/or group studies that appear to demonstrate a dissociation between these different forms of memories have attracted much theoretical discussion. For instance, the seemingly preserved semantic memory coupled with the severely impaired episodic memory reported in cases of selective hypoxic damage to the hippocampus in childhood (e.g., Vargha‐Khadem et al., 1997) are at odds with the form of hippocampal‐equivalence proposed by the SC model. Moreover, reports of spared semantic memory within a context of severely impaired episodic memory in patients with adult‐onset MTL/HC amnesia (e.g., Rosenbaum et al., 2008; Verfaellie, Koseff, & Alexander, 2000) are difficult to accommodate within such models. However, the structural selectivity of these dissociations have been fiercely disputed and counter‐findings are frequently presented (e.g., Manns, Hopkins, & Squire, 2003; Reed & Squire, 1998; but see Winocur & Moscovitch, 2011). Moreover, uncertainties surrounding a clear division between episodic and semantic memory are evident in the semantic dementia literature (e.g., Burianova, McIntosh, & Grady, 2010; Greenberg & Verfaellie, 2010).

Further divisions within the field also exist between so‐called dual‐process memory theorists (for reviews see Aggleton & Brown, 2006; Eichenbaum, Yonelinas, & Ranganath, 2007; Gardiner & Java, 1993; Montaldi & Mayes, 2010; Yonelinas, 2002) and single‐process memory theorists (for reviews see Clark, 2018; Squire, Wixted, & Clark, 2007; Wixted, 2007). In essence, dual‐process theories argue that the extended hippocampus (i.e., the hippocampus, the anterior nucleus of the thalamus, and the mamillothalamic tract) is selectively involved in recollection‐based memory processes, while familiarity‐based recognition is supported by the perirhinal memory system (i.e., the perirhinal cortex and mediodorsal nucleus). Recollection‐based memory is defined as memory with an associated subjective feeling of remembering, which encompasses both free recall and event recognition if that recognition is accompanied by the full recall of the event and encoding context. Familiarity‐based recognition is recognition that occurs with an isolated sense of familiarity and without the recollection of the contextual details present at acquisition. Findings consistent with this viewpoint offer evidence of specific recollection deficits in patients with selective damage to the hippocampal memory system (e.g., Brandt, Gardiner, Vargha‐Khadem, Baddeley, & Mishkin, 2009; Holdstock et al., 2002) and/or a double dissociation between recall‐ and familiarity‐based recognition (e.g., Bowles et al., 2010; see also Brandt, Eysenck, Nielsen, & von Oertzen, 2016). However, single‐process theorists dispute these functional and structural dissociations, arguing that these apparent dissociations are driven by differences in memory strength, and point to studies that report impairment of both recall and recognition (e.g., Manns et al., 2003), and of both the recollection and familiarity‐based recognition (for review see Wixted & Squire, 2010), following bilateral hippocampal damage.

The extent of the conflict in the aforementioned studies is striking, not least due to the fact that many have been observed using standardized neuropsychological tasks such as the Autobiographical Memory Interview (AMI) (Kopelman, Wilson, & Baddeley, 1990) and the Doors and People Test (D&P) (Baddeley & Nimmo‐Smith, 1994). Standardized memory tasks, if administered correctly, eliminate variation in administration and scoring methodology as a source of these differences. Moreover, the D&P test equates the difficulty levels of the recall and recognition subtests—a control that counters the memory strength hypothesis proposed by single theorists to explain apparent recall/recognition dissociations in the literature (e.g., Dunn, 2008). Hence, reconciling these theoretical stalemates using identical neuropsychological methods has been largely unsuccessful. Moreover, while novel neuroimaging techniques such as the combination of high‐resolution structural and functional magnetic resonance imaging and advanced analytical methods (such as multivoxel pattern analysis) can undoubtedly add unique leverage on these issues (for review see Maguire, 2014), traditional neuropsychological approaches still play an important role in the resolution of these conflicts.

One persistent and major challenge for those undertaking such studies is that patients with selective and uniform hippocampal damage are exceedingly rare. Hence, group studies with hippocampal amnesic patients typically utilize patients with damage acquired through a range of diverse etiologies (e.g., cardiac arrest, carbon monoxide poisoning, drug overdose, or “unknown,” Manns et al., 2003) or include patients with varying neuropathologies including damage to the hippocampus, frontal lobe, and thalamus (e.g., Manns & Squire, 2002). These studies therefore assume a uniformity across patients that is largely unsubstantiated, as studies combining both neuropsychological observations and postmortem descriptions are understandably rare (for notable exceptions see Zola‐Morgan, Squire, & Amaral, 1986; Rempel‐Clower, Zola, Squire, & Amaral, 1996; Annese et al., 2014), and determining hippocampal functionality on the basis of structural MRI can be misleading (Mullally, Hassabis, & Maguire, 2012).

In this study, we assess a homogenous population with a highly discrete hippocampal lesion that is clinically stable after an acute phase: patients with voltage‐gated potassium channel complex antibody related limbic encephalitis (VGKCC‐Ab LE) (Finke et al., 2017; Miller et al., 2017). VGKCC‐Ab LE is a rare autoimmune condition discovered in 2004 with a prevalence of about 1 in 400,000 (Vincent et al., 2004). It is an autoimmune inflammatory disorder that causes long‐term memory impairment, seizures, and sometimes‐behavioral disturbances in its acute phase, but patients recover and can be left with selective memory deficits (Buckley et al., 2001). Patients who test positive for VGKCC antibodies are further subdivided into those with anti‐leucine‐rich glioma inactivated (anti‐LGI‐1) encephalitis (who present with limbic symptoms), anti‐contactin‐associated protein‐like 2 (anti CASPR‐2) (who present with both central and peripheral symptoms) and a third group who do not have antibodies against LGI‐1 or CASPR‐2 and who present with heterogeneous symptoms (Bastiaansen, van Sonderen, & Titulaer, 2017). Both LGI‐1 and CASPR‐2 are different, well‐described clinical phenotypes (Van Sonderen, Petit‐Pedrol, Dalmau, & Titulaer, 2017), with the former specific for the hippocampus (van Sonderen et al., 2016). Here we consider patients with this brain phenotype as a unique opportunity to test the effect of an anatomically selective hippocampal lesion. Hence, unlike the previously discussed neuropsychological studies of patients with selective hippocampal damage, we utilized a uniform cohort of stable patients to adjudicate between the entrenched and conflicting theoretical perspectives described earlier. In total, we tested seven patients (two female, mean age: 66 years, range: 51–70 years) with VGKCC‐Ab LE recruited via the Cognitive Clinic at the Royal Victoria Infirmary, Newcastle upon Tyne, United Kingdom. Patients were selected if they had positive VGKCC antibody level of >1000pM at the time of diagnosis and a clinical phenotype consistent with LGI‐1 limbic encephalitis after review by a Cognitive Neurologist (see Table S1 for further details).

At presentation in the acute phase of illness, MR brain imaging showed increased hippocampal signal intensity on T2 or FLAIR sequences in five out of seven patients (Figure 1: Acute Phase). Subsequently, in the stable chronic phase and more than 1 year after acute presentation, all patients had additional structural MRI (see Data S1). This revealed hippocampal atrophy in each patient that was specific to hippocampal, as opposed to parahippocampal structures (Figure 1: Stable Phase). An extensive neuropsychological assessment (see Table S2) was also performed within 2 months of patients undergoing stable phase structural MRI. Results were consistent with a selective memory impairment as opposed to a global cognitive insult.

**Figure 1:**
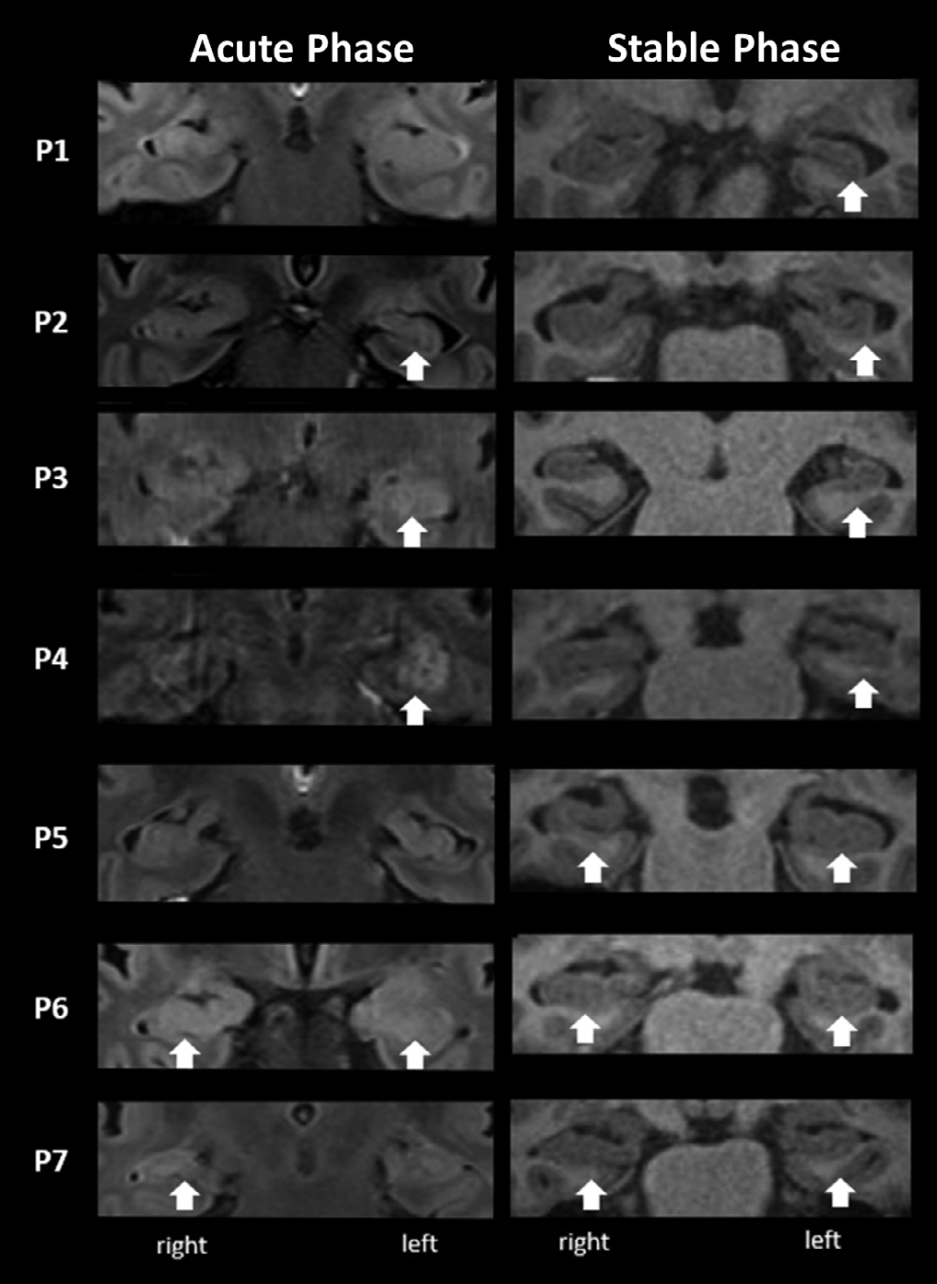
Structure MRI of patients from initial presentation in the acute phase (T2 coronal FLAIR; left column) and at time of neuropsychological testing in the acute phase (T1 coronal; right column). White arrows indicate increased signal intensity in the left column and subsequent atrophy in the right column (as reported by neuroradiologists)

To test the outstanding issues outlined earlier, we administered two further standardized neuropsychological measures—the AMI and the D&P test (Baddeley & Nimmo‐Smith, 1994). The AMI is a standardized test of autobiographical and personal semantic memories that uses a structured interview to assess memory across three different time‐periods (i.e., recent events: within the last year; early adulthood: 19–29 years old; and childhood: up until 18 years of age). While the AMI is argued to lack the sensitivity at detecting remote memory loss relative to tasks such as the autobiographical interview (Levine, Svoboda, Hay, Winocur, & Moscovitch, 2002), it has the advantage of available standardized scores and a long‐standing, ubiquitous presence within this literature. The D&P assesses verbal and visual recall and recognition. Critically, it also equates the difficulty levels of the recall and recognition subsets. Both the AMI and the D&P tasks have been used extensively in the hippocampal literature enabling direct comparison with previous work.

With respect to the AMI, we hypothesized that if the hippocampus plays a time‐limited role in memory retrieval, then VGKCC‐Ab LE patients should show impairment on recall of recent autobiographical memories but a preservation of remote memories. However, if retrograde memory deficits are evident across the lifespan (and are therefore nongraded), then this results would favor alternative theories, such as Multiple Trace Theory (MTT; Nadel & Moscovitch, 1997), Transformation Theory (Winocur & Moscovitch, 2011), and Scene Construction Theory (Maguire & Mullally, 2013), that do not draw this distinction. Similarly, the AMI also enabled us to assess whether VGKCC‐Ab LE patients would be equally impaired on autobiographical and personal semantic memory (as predicted by the standard consolidation model), or show greater impairment to autobiographical memories (as predicted by the aforementioned alternative theories).

As anticipated, the LE patients demonstrated a striking impairment for autobiographical material, with six of the seven patients showing definite impairment in the recall of recent autobiographical memories (Figure 2a). Moreover, their performance at a group level fell well below the range observed in health controls (Kopelman et al., 1990; Figure 2b). However, this clear impairment was coupled with a robust preservation of personal semantic memories at each of these time points (Figure 2c,d). This dissociation is consistent with the recently reported dissociation between episodic and semantic memory impairment/preservation observed in a group of 16 LGI1‐VGKCC‐Ab LE patients (Miller et al., 2017). Moreover, at the group level, and contrary to the standard consolidation model, our VGKCC‐Ab LE patients demonstrated no temporal‐gradient when recalling past personal memories. More specifically, no significant within‐group differences were observed between recent and early life autobiographical memories (t[6] = −0.367, *p* = .726), between early life and childhood autobiographical memories (t[6] = −1.131, *p* = .301), and between recent life and childhood autobiographical memories (t[6] = −1.686, *p* = .143) (Figure 2b). The same absence of a temporal‐gradient was evident in personal semantic memories (t(6) = 0.977, *p* = .366; t(6) = 0.803, *p* = .452; t(6) = 0.314, *p* = .764; Figure 2d).

**Figure 2:**
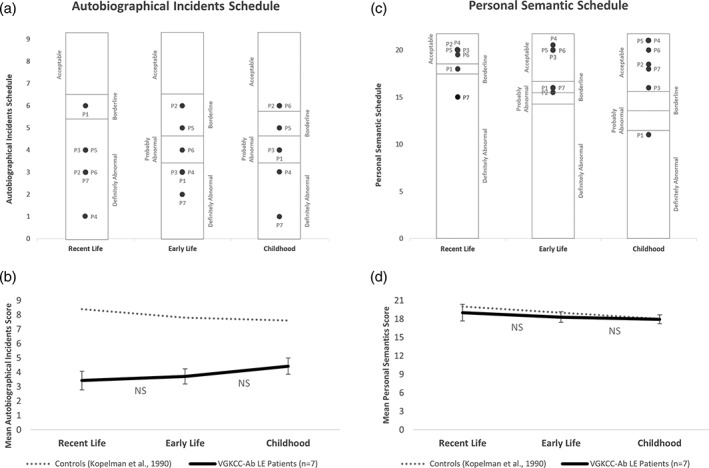
The autobiographical memory interview (AMI). (a) Autobiographical incidents schedule—individual patient data. VGKCC‐Ab LE patient scores are represented relative to the cutoff points for healthy controls cited in Kopelman et al. (1990): “Acceptable”: ±1 *SD* of the control mean; “borderline”: Between 1 *SD* and 2 *SD* below the control mean; “probably abnormal”: > 2 *SD* below the control mean; “definitely impaired”: Scores at or below which none of the healthy controls scored. (b) Autobiographical incidents schedule—group data. NS, nonsignificant differences between epochs. (c) Personal semantic schedule—individual patient data. Cutoff points as per (a). (d) Personal semantic schedule—group data

At an individual level, the pattern of impairment of autobiographical memory across each life epoch was more mixed; with two of the seven patients reporting autobiographical memories for their childhood that fell within an acceptable range (Figure 2a). This pattern of temporally graded retrograde amnesia has previously been observed in a patient with presumed autoimmune LE associated with the human herpes virus 6 (Kapur & Brooks, 1999) and presumed autoimmune LE following systemic lupus erythematosus (Schnider, Bassetti, Schnider, Gutbrod, & Ozdoba, 1995), although these conditions may not preferentially target the hippocampus. In both of the VGKCC‐Ab LE cases reported here however, the seemingly acceptable childhood autobiographical memory performance resides on the lower boundary of acceptable category. Moreover, the underlying reason for these qualitative (but nonsignificant quantitative) differences is unclear. One possibility (in line with MTT and Transformation theory) is that these acceptable childhood autobiographical memories may be disproportionally benefitting from well‐rehearsed personal semantic childhood knowledge that are clearly intact in this group (see Figure 2c,d). Without the benefit of more nuanced measures (such as the use of the autobiographical interview to explore recollections from each of these discrete time points), this modest but nonsignificant benefit for childhood autobiographical memories remains unclear. Overall, however these findings raise important challenges for standard consolidation model.

With respect to the D&P, we asked whether VGKCC‐Ab LE patients would demonstrate a uniform impairment across the recall and recognition subtests (consistent with a single‐process theories) or whether they would display a dissociation (i.e., consistent with a dual‐process theories). VGKCC‐Ab LE patients performed significantly worse than age‐ and gender‐matched matched controls (*n* = 14: two/patient, four female, mean age: 65 years, range: 52–73) on immediate verbal recall (*U* = 2.0, *p* < .001), delayed verbal recall (*U* = 20.0, *p* = .031), and delayed visual recall (*U* = 22.0, *p* = .046), but not on immediate visual recall (*U* = 36.5, *p* = .360). In contrast, no significant deficits in either verbal recognition (*df* = 19, *t* = −0.288, *p* = .777) and visual recognition (*df* = 19, *t* = 0.645, *p* = .527) memory were observed in the patient group relative to the matched controls (Figure 3a), and there were no differences when performance on the easy (i.e., Set A) and the hard (i.e., Set B) recognition trials were compared between patients and controls in either the verbal or the visual domains (Verbal Recognition Set A [*U* = 20.5, *p* = .161], Verbal Recognition Set B [*df* = 15, *t* = 0.539, *p* = .598], Visual Recognition Set A [*U* = 26.0, *p* = .417], Visual Recognition Set B [*df* = 15, *t* = 0.501, *p* = .623] [Figure 3b]). Moreover, recall memory was significantly lower than recognition memory in patients (U = 14.0, *p* = .007; Figure 3c). No verbal/visual discrepancy was observed (*df* = 15, *t* = 1.580, *p* = .131; Figure 3c).

**Figure 3:**
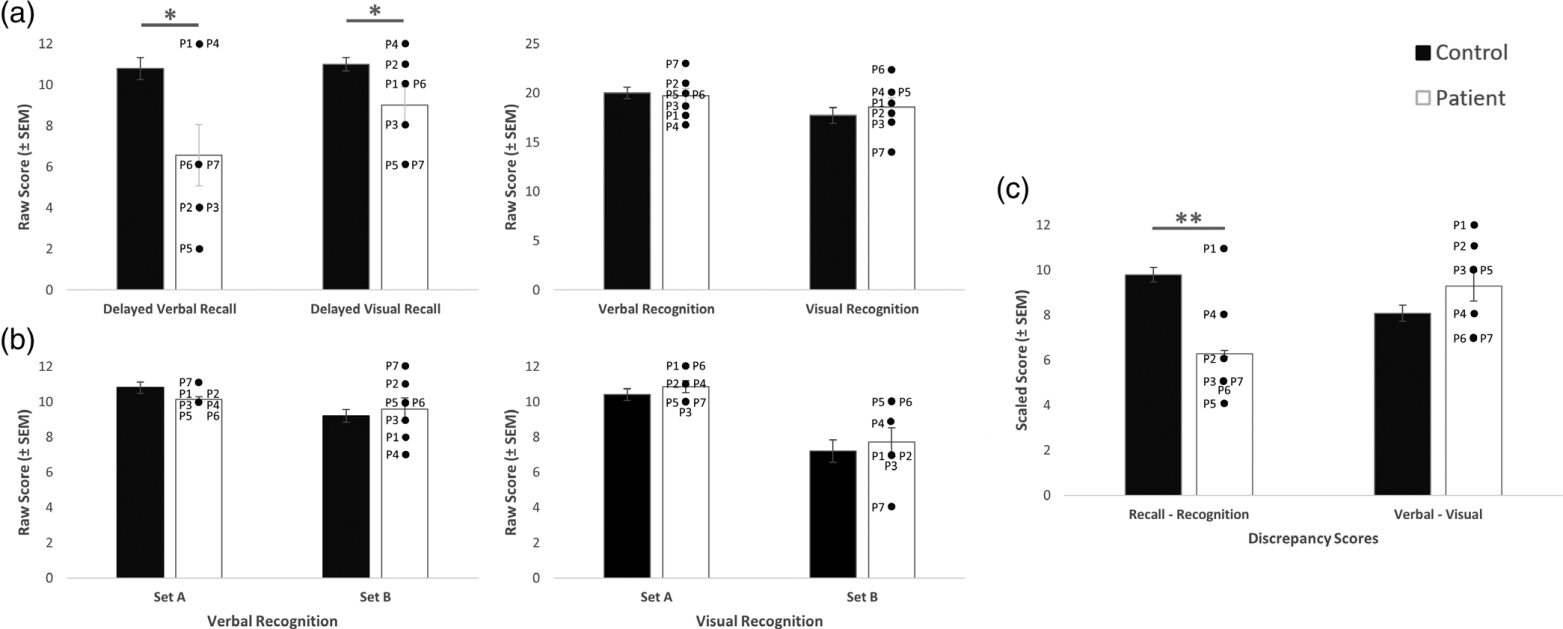
The doors and people task. Bar graphs representing patient and matched‐control mean [± *SEM*] and individual patient scores in (a) verbal and visual delayed recall memory, and verbal and visual recognition memory; (b) verbal recognition memory—set A (easy) and set B (hard) and visual recognition memory—Set A (easy) and set B (hard); and (c) Overall “Recall—Recognition” discrepancy score and “Verbal—Visual” discrepancy score. * *p* < .005; ** *p* < .001

Hence, in this study we explored three key areas of controversy in the memory literature, that is, whether damage to the human hippocampus selectively impairs recent versus remote memories, autobiographical versus personal semantic memories, and recall versus recognition memory, in a stable patient population that provide a novel lesion model to test different hypotheses for hippocampal function. Further work is now necessary to explore additional hippocampal‐based processes with this patient group utilizing for instance, cognitive paradigms that seek to disentangle the more nuanced, and perhaps overlapping, components recollection and familiarity‐based recall (e.g., Sadeh, Moran, Stern, & Goshen‐Gottstein, 2018), or further exploring the increased pattern of variability observed in remote episodic memory recall. In addition, more studies are needed to see if in‐vivo imaging findings correlate with discrete cognitive markers of impaired hippocampal function. A recent study by Butler and colleagues (Loane et al., 2019), investigated both the structural and functional whole‐brain abnormalities in a mixed cohort of 24 LGI1 and CASPR2 memory impaired VGKCC‐Ab LE patients. Alongside focal hippocampal atrophy, they observed atrophy in the mediodorsal thalamus that correlated strongly with hippocampal volume reductions, in addition to volume reduction in the posteromedial cortex. These structural differences, indicative of “network‐specific degeneration across the hippocampal‐diencephalic‐cingulate circuitry” (pp. 8), did not however correlate with residual memory performance. Memory performance instead correlated with measures of both interhippocampal and posteromedial cortico‐hippocampal functional connectivity. Hence, this increasingly well‐characterized group of patients may represent an important neuropsychological model of disrupted functional connectivity whose critical locus resides within the hippocampus. This is broadly consistent with other studies who have reported selective hippocampal damage in VCKCC_Ab LE patients, perhaps even at the level of the hippocampal subfields (Miller et al., 2017). As such, future neuropsychological studies with VCKCC_Ab LE patient cohorts, in combination with studies utilizing high‐resolution structural and functional MRI and advanced analytical methods, could offer important traction on many of the entrenched impasses firmly rooted in the hippocampal literature.

## Supporting information


**Appendix S1** : Supporting Information
